# Roles of Hostility and Depression in the Association between the MAOA Gene Polymorphism and Internet Gaming Disorder

**DOI:** 10.3390/ijerph18136910

**Published:** 2021-06-27

**Authors:** Ju-Yu Yen, Wei-Po Chou, Huang-Chi Lin, Hung-Chi Wu, Wen-Xiang Tsai, Chih-Hung Ko

**Affiliations:** 1 Department of Psychiatry, Kaohsiung Municipal Ta-Tung Siaogang Hospital, Kaohsiung Medical University, Kaohsiung City 812, Taiwan; yenjuyu@cc.kmu.edu.tw; 2 Department of Psychiatry, Faculty of Medicine, College of Medicine, Kaohsiung Medical University, Kaohsiung City 807, Taiwan; cochigi@yahoo.com.tw; 3 Department of Psychiatry, Kaohsiung Medical University Hospital, Kaohsiung Medical University, Kaohsiung City 807, Taiwan; webber1007@gmail.com (W.-P.C.); thunderracco@gmail.com (W.-X.T.); 4 Department of Addiction Science, Kai-Syuan Psychiatric Hospital, Kaohsiung City 802, Taiwan; wuhcmail@gmail.com; 5 Department of Psychiatry, Kaohsiung Municipal Siaogang Hospital, Kaohsiung Medical University, Kaohsiung 801, Taiwan

**Keywords:** MAOA EcoRV polymorphism, hostility, bioamine, internet gaming disorder

## Abstract

The metabolism of bioamine in the central nervous system contributes to the development of addiction. We examined the roles of hostility and depression in the association between internet gaming disorder (IGD) and monoamine oxidase-A (MAOA) EcoRV polymorphism (rs1137070). A total of 69 adults with IGD and 138 without IGD were recruited through diagnostic interviewing. We evaluated participants for rs1137070, depression, and hostility. The participants with the TT genotype of rs1137070 had a higher odds ratio of 2.52 (1.37–4.64) for IGD compared with the C carriers. Expressive hostility behavior and hostility cognition mediated the association between rs1137070 and IGD. Indicating lower MAOA activity, the TT genotype predicted IGD and higher expressive hostility behavior and hostility cognition. Expressive hostility behavior and hostility cognition may underline the association between rs1137070 and IGD. Assessment of and intervention for hostility behavior and cognition should be provided to attenuate the risk of IGD, particularly in those with the TT genotype. Further brain imaging or neurobiological studies are required to elucidate the possible mechanism underlying the association between MAOA activity and IGD.

## 1. Introduction

The fifth edition of the *Diagnostic and Statistical Manual of Mental Disorders* (DSM-5), published in 2003, includes internet gaming disorder (IGD). In 2019, the *11th Revision of the International Classification of Diseases* categorized gaming disorder (GD) as an addictive disorder. IGD results in impairments in daily functioning such as career, academic, job, and social functions [1], and deserves further evaluation and intervention. Considering the heterogeneity of IGD, a personalized intervention to meet individual patient preferences is essential. However, studies determining the genetic factors of GD for a designed intervention are scarce. Until now, only two studies have demonstrated associations of the C allele of rs1044396 in the nicotinic acetylcholine receptor alpha 4 subunit (CHRNA4) [2] and rs2229910 of NTRK3 [3] with IGD. Further research to evaluate the associations between polymorphisms and their associated factors is necessary for intervention design.

### 1.1. Depression, Hostility, and IGD

Depression is frequently reported to be associated with IGD [4]. A prospective study suggests that IGD worsens depression symptoms [5]. A meta-analysis demonstrated that one in three individuals with IGD have depression [4]. Because violent content is popular in online gaming, numerous studies have evaluated the association between aggression and IGD to determine the effects of violence in media [6]. The association between aggression and IGD has been frequently reported in adults [7] and adolescents [8]. Aggression is considered an antecedent of GD [9], and it impedes recovery from IGD [10]. These results suggest that addressing aggression and depression can play a significant role in recovery from IGD.

### 1.2. Monoamine Oxidase Polymorphism, Depression, and Aggression

Monoamine oxidase (MAO) catalyzes the oxidative deamination of many biogenic amines. MAO is essential for regulating synaptic concentrations of serotonin, dopamine, norepinephrine, and other catecholaminergic neurotransmitters in the central nervous system. The monoamine oxidase A gene (MAOA gene) has been mapped to the short arm of the X chromosome; thus, functional polymorphisms of this locus are expected to manifest in a sex-specific fashion. The genetic variation in MAOA contributes to impulsive aggression [11] and depression [12] through a neurobiological mechanism. Higher MAOA activity can result in the rapid catalyzation of serotonin and norepinephrine, thereby contributing to depression [13]. Furthermore, lower MAOA activity is reported to be associated with impulsive aggressive behavior [11]. However, depression and hostility are both associated with IGD. Evaluating the associations involving MAOA polymorphism may highlight the mechanism of IGD.

A previous study demonstrated that individuals with the C allele of the MAOA EcoRV polymorphism (rs1137070; 1460C > T polymorphism in exon 14) had higher MAOA activity [14]. The low MAOA low activity variant is reported to increase susceptibility in processing threat-related emotions [15]. The T frequency of rs1137070 ranges from 50.9% to 63.5% [15,16]. A meta-analysis demonstrated that the T allele associates with major psychiatric disorders, such as depression, bipolar, and schizophrenia [17]. Furthermore, the T allele of rs1137070 is reportedly associated with smoking [16]. These results suggest that rs1137070 affects addiction risk through its effect on MAOA activity and is sensitive to emotions such as depression or irritability. However, studies have overlooked the association between rs1137070 and IGD.

We hypothesize that rs1137070 contributes to the development of IGD. This association may result from the effect on aggressive behavior and depression. Accordingly, this study evaluated the associations among rs1137070, hostility, depression, and GD. If an association was significant, this study further reveals the confounding effect of hostility or depression in the association between rs1137070 and GD.

## 2. Methods

### 2.1. Participants

Participants with IGD (IGD group), matched regular gamers (RG group), and non-gamers (NG group) were recruited through advertisement postings on the online bulletin boards of universities. The participants of the IGD group were aged 20–38 years, had >12 years of education, played online video games for ≥4 h per day on weekdays and ≥6 h per day on weekends, and maintained a consistent pattern of internet gaming for over 2 years. This hour threshold indicates that gaming occupied half or more of available time in daily life as a previous study [7]. We then confirmed their diagnosis through a diagnostic interview according to the IGD criteria of the DSM-5.

The RG and NG group participants were frequency matched by sex and age (±3 years) with the IGD group participants. In the NG group, we included individuals making nonessential use of the Internet for <4 h per day [18], which excluded regular gaming. In the RG group, we recruited individuals who participated in regular online gaming (≥3 days per week) without fulfilling the diagnostic criteria of IGD. The RG and NG group were combined to be the control group. The diagnoses of participants were confirmed through psychiatric interviews, which comprised three parts:

1. diagnostic interviews for the IGD group based on the DSM-5 criteria;
2. a Chinese version of the Mini International Neuropsychiatric Interview [19] to exclude those with psychotic disorders, bipolar I disorder, and substance use disorders;
3. an interview to note their history to exclude those with intellectual disabilities, severe physical disorders, or brain injury.

In total, 69 participants were included in the IGD group and 138 in the control group (69 each in the RG and NG groups) after obtaining their informed consent. The study was approved by the Institutional Review Board of Kaohsiung Medical University Hospital, Taiwan (KMUHIRB-SV(II)-20150081).

### 2.2. Measures

#### 2.2.1. DSM-5 Diagnostic Criteria for IGD

We prepared a semi-structured interview to examine the severity and frequency of each DSM-5 criterion for the IGD group. Participants fulfilling five or more criteria were placed in the IGD group.

***Center for Epidemiological Studies’ Depression Scale (CES-D)***. The 20-item Mandarin Chinese version [20] of the CES-D [21] is a self-administered evaluation assessing participants’ frequency of depressive symptoms over the past week. The Cronbach’s α of the CES-D in this study was 0.78. The scale was utilized to evaluate depression.

***The Buss–Durkee Hostility Inventory-Chinese Version-Short Form (BDHIC-SF)***. The 20-item, 5-point Likert-type BDHIC-SF assessed the four dimensions of hostility construct: hostility cognition, hostility affection, expressive hostility behavior, and suppressive hostility behavior. The Cronbach’s α was 0.93, and the 4-week test–retest reliability was 0.80. Higher scores indicated higher irritability [22].

#### 2.2.2. Genotyping of rs1137070

In this study, we used the QIAamp DNA Blood Mini Kit (Qiagen, Hilden, Germany) to obtain the total genomic DNA (gDNA) from peripheral lymphocytes. The genotyping was performed using a 10 μL mixture containing 1 μL of genomic DNA (30 ng/μL), 5 μL of TaqMan Universal PCR Master Mix (Applied Biosystems, Nieuwerkerk a/d Ijssel, the Netherlands), 0.5 μL of 20 × SNP assay mix (Taqman assay: assay ID of MAOA rs1137070: C_8878813_20; Applied Biosystems), and 3.5 μL of double-distilled H_2_O. Amplification was performed using the AB 7900HT fast real time PCR system for 10 min at 95 °C, followed by 45 cycles of 15 s each at 92 °C and 60 s each at 60 °C. Genotypes were scored using the algorithm and software supplied by Applied Biosystems.

### 2.3. Procedure

All participants completed the diagnostic interview, blood test, and questionnaire assessment simultaneously during the daytime. They were prevented from smoking or consuming alcohol two hours before the test.

### 2.4. Statistical Analysis

We used the independent *t* test to evaluate differences in the *CES-D* and *BDHIC-SF* scores between the IGD and control groups. The association between IGD and rs1137070 was analyzed using the chi-square test. In addition, the independent *t* test was used to evaluate differences in the *CES-D* and *BDHIC-SF* scores between genotypes of rs1137070 (TT versus C carriers). The odds ratio (OR) for rs1137070 (TT versus C carriers) in the IGD group relative to the control group was determined using logistic regression analysis after controlling for sex and age. Next, we regressed IGD on rs1137070 and *BDHIC-SF* scores to test its mediating effect on the association between IGD and rs1137070 [23]. A *p*-value of <0.05 was considered significant in all analyses performed using SPSS (version 26.0; IBM, Armonk, NY, United States).

## 3. Results

No difference was observed in sex and age between the IGD group and controls.

### 3.1. Association between IGD and Hostility

The independent *t* test (Table 1) revealed that the IGD group scored higher in depression (*t* = 9.38, *p* < 0.001), expressive hostility behavior (*t* = 6.29, *p* < 0.001), hostility cognition (*t* = 7.83, *p* < 0.001), hostility affect (*t* = 7.01, *p* < 0.001), suppressive hostility (*t* = 6.08, *p* < 0.001), and hostility total score (*t* = 9.81, *p* < 0.001).

### 3.2. Association between rs1137070 and Hostility

The *t* test results indicated that the participants with the TT genotype of rs1137070 had higher expressive hostility behavior (*t* = 2.39, *p* = 0.018), hostility cognition (*t* = 2.10, *p* = 0.037), and hostility total (*t* = 2.17, *p =* 0.031) scores than the C carriers (Table 2). The results thus revealed that the participants with the TT genotype were more hostile. However, no difference was observed in depression between individuals with the TT genotype and C carrier.

### 3.3. Association between IGD and rs1137070

The chi-square test demonstrated that the diagnosis of IGD was significantly associated with rs1137070 (χ^2^ = 8.70, *p* = 0.003; Table 1). Forward logistic regression analysis revealed that after controlling for sex and age, compared with the C genotype carriers, the participants with the TT genotype had a higher OR of being diagnosed as having IGD (OR = 2.52, 95% confidence interval (CI) = 1.37–4.64, *p* = 0.003; Table 3, model 1). Therefore, the participants with the TT genotype were more likely to develop IGD.

As indicated in Table 1 and Table 2, expressive hostility behavior and hostility cognition were associated with IGD and the TT genotype. Thus, expressive hostility behavior and hostility cognition were controlled for in the regression model of MAOA polymorphism and IGD to test their mediating effect. Controlling for expressive hostility behavior and hostility cognition, the OR for IGD in the participants with the TT genotype was minimized and was significant (OR = 2.06, 95% CI = 1.002–4.234, *p* = 0.049; Table 3, model 2). In the forward regression model, expressive behavior (OR = 1.16, 95% CI = 1.06–1.26, *p* = 0.001) and hostility cognition (OR = 1.256, 95% CI = 1.14–1.36, *p* < 0.001) were significantly associated with IGD. Thus, the results suggest that expressive behavior and hostility cognition mediate the association between MAOA polymorphism and IGD.

## 4. Discussion

This study demonstrated that individuals with the TT genotype of rs1137070 had 2.52 times the OR for IGD relative to those with the C allele. Despite the robust significant association, interpretations should be made with caution considering the small sample size. The T allele of rs1137070 is associated with smoking behavior [15,24]. Our results may indicate that the TT genotype is associated with addictive behavior. A previous study demonstrated that the T allele of rs1137070 is associated with major psychiatric disorders, such as schizophrenia, depression, and others [16]. However, the reasons for the association between the T allele and psychiatric disorders remain unclear as opposed to its association with addictive behavior. Evaluating the effect of polymorphism on associated psychological and behavioral factors may elucidate the mechanism of IGD.

IGD was associated with depression and hostility. We hypothesized that MAOA would associate with depression. However, no significant association was noted. Although individuals with the TT genotype scored higher in depression, the finding was not significant. A previous study demonstrated that the TT genotype of rs1137070 was associated with depression in females with only marginal significance in males [17]. In this study, most participants (77.3%) were male. This may contribute to the nonsignificant association between the TT genotype and depression in this study. Furthermore, the small sample may have limited the power to demonstrate the difference.

The TT genotype of rs1137070 indicated lower MAOA activity in the study by Tu et al. [14]. A previous study demonstrated that low activity of the MAOA polymorphism is associated with aggression among socially excluded individuals [25]. Furthermore, lower brain MAOA activity in positron emission tomography is reported to predict aggression [26]. Low MAOA activity is suggested to contribute to aggressive behavior [27]. In line with a previous study, this study demonstrated that individuals with the TT genotype have higher expressive hostility behavior and hostility cognition than C carriers. This result demonstrated the association between rs1137070 and aggressive behavior and hostility cognition. This result indicated that the TT genotype is associated with hostility cognition and aggressive behavior. This effect may partly support the association between the T allele and schizophrenia, in which persecutory delusion and hostility can occur under psychosis. However, the reasons why low MAOA activity contributes to aggressive behavior deserve further study, for example, a PET study can be conducted to determine MAOA activity and its effect on cognition and behavior.

The TT genotype was associated with both hostility and IGD in this study. Hostility was also associated with IGD. The logistic regression demonstrated that the IGD OR for those with the TT genotype decreased from 2.5 (*p* = 0.003) to 2.06 (*p* = 0.0493) after controlling for hostility. Based on the concept of Baron and Kenny [23], expressive hostility behavior and hostility cognition mediated the association between the TT genotype and IGD.

Considering that the genotype was unchangeable before IGD developed, this result may suggest that individuals with the TT allele of rs1137070 are more likely to develop IGD and are more hostile. Furthermore, individuals with IGD have higher hostility, thereby impeding recovery from IGD [10]. Thus, effective interventions for hostility, such as cognitive behavior therapy [28] or mindfulness [29], should be provided to individuals with IGD. For those with IGD and the TT genotype of rs1137070, expressive hostility behavior and hostility cognition should be assessed and addressed. Finally, individuals with rs1137070 should be assessed for their hostility and interventions provided to decrease their risk of IGD.

## 5. Limitations

Three limitations may affect result interpretation. First, because the sample size was small in this presenting study, the effects of rs1137070 on hostility and IGD should be interpreted with caution. Further, these effects should be proved in further study with an adequate number of samples, such as about 800 participants [30]. Second, the cross-sectional design of this study makes it difficult to make a conclusion regarding the causal relationship between hostility and IGD. Third, many other polymorphisms such as MAOA-uVNT and MAOA *Rnu*4HI may play an important role in MAOA activity [31]. The effects of these polymorphisms on IGD should be evaluated together in a future study.

## 6. Conclusions

Individuals with the TT genotype of rs1137070 were more likely to develop IGD, as they had higher expressive hostility behavior and hostility cognition. Expressive hostility behavior and hostility cognition mediated the association between rs1137070 and IGD. Hostility behavior and cognition should be assessed and addressed in individuals with IGD, particularly those with the rs1137070 TT genotype. The mechanism underlining this association should be evaluated through brain imaging or a neurobiological study in the future.

## Figures and Tables

**Table 1:** Age, sex, depression, and hostility scores, and MAOA EcoRV polymorphism of the IGD group and controls.

	IGD Mean (SD)	Control Mean (SD)	*t*
Age	25.32 (4.20)	25.73 (3.78)	−0.71
Depression ^a^	23.91 (8.62)	12.58 (7.98)	9.38 ***
Expressive hostility behavior ^b^	17.75 (4.41)	13.95 (3.94)	6.29 ***
Hostility cognition ^b^	20.99 (4.60)	15.92 (4.28)	7.83 ***
Hostility affect ^b^	11.72 (4.17)	8.14 (3.05)	7.02 ***
Suppressive hostility ^b^	17.41 (4.18)	13.70 (4.11)	6.08 ***
Hostility total ^b^	67.87 (11.78)	51.72 (10.84)	9.82 ***
	IGD *n* (%)	Control *n* (%)	χ^2^
Gender			
Male	54 (78.30%)	108 (78.30%)	0.00
Female	15 (21.70%)	30 (21.70%)	
MAOA polymorphism			
TT/T0	44 (63.80%)	58 (42.00%)	8.70 ***
C carrier	25 (36.20%)	80 (58.00%)	

*** *p* < 0.001. ^a^ Depression: score in the CES-D. ^b^ Hostility: score in the BDHIC-SF

**Table 2:** Associations among depression and hostility scores and the MAOA EcoRV polymorphism.

MAOA Polymorphism	TT *Mean* (SD)	C Carrier *Mean* (SD)	*t*
CESD ^a^	16.79 (9.85)	15.93 (9.73)	0.63
Expressive hostility ^b^ behavior ^b^	15.96 (4.80)	14.50 (4.02)	2.39 *
Hostility cognition ^b^	18.34 (5.13)	16.90 (4.77)	2.10 *
Hostility affect ^b^	9.72 (4.07)	14.75 (4.50)	1.40
Suppressive hostility ^b^	15.13 (4.47)	14.75 (4.50)	0.60
Hostility total ^b^	59.15 (13.68)	55.11 (13.08)	2.17 *

* *p* < 0.05. ^a^ Depression: score in the CES-D. ^b^ Hostility: score in the BDHIC-SF.

**Table 3:** Hierarchical logistic regression evaluated the association between IGD and the MAOA polymorphism after controlling for expressive hostility behavior and hostility cognition.

IGD Versus Controls	Wald X^2^	df	Exp(β)	95% CI
Model 1				
Age	0.47	1	0.97	0.90–1.05
Gender	0.25	1	0.83	0.39–1.73
MAOA polymorphism	8.76 **	1	2.52	1.37–4.64
Model 2				
Age	0.06	1	1.01	0.92–1.11
Gender	1.11	1	0.62	0.25–1.51
MAOA polymorphism	3.86 *	1	2.06	1.00–4.23
Expressive hostility behavior ^a^	10.40 **	1	1.16	1.06–1.26
Hostility cognition ^a^	23.45 ***	1	1.25	1.14–1.36

* *p* < 0.05; ** *p* < 0.01; *** *p* < 0.001. ^a^ Hostility: score in the BDHIC-SF.

## Data Availability

The data presented in this study are available on request from the corresponding author. The data are not publicly available due to privacy.
